# Rapid serological detection of autoantibodies associated with Sjögren's syndrome

**DOI:** 10.1186/1479-5876-7-83

**Published:** 2009-09-24

**Authors:** Peter D Burbelo, Kathryn H Ching, Alexandra T Issa, Caroline M Loftus, Yi Li, Minoru Satoh, Westley H Reeves, Michael J Iadarola

**Affiliations:** 1 Neurobiology and Pain Therapeutics Section, Laboratory of Sensory Biology, National Institute of Craniofacial Research, National Institutes of Health, Bethesda, Maryland, USA; 2 Division of Rheumatology and Clinical Immunology and Center for Autoimmune Diseases, University of Florida, Gainesville, Florida, USA

## Abstract

**Background:**

Sjögren's syndrome (SjS) is a relatively common autoimmune disease characterized by oral and ocular dryness. There is an increasing need for simple, sensitive and rapid technologies for the diagnosis of SjS and other autoimmune diseases. Here we investigated whether a quick version of luciferase immunoprecipitation systems (QLIPS) could be used to produce a rapid, specific and quantitative test to detect autoantibodies associated with SjS.

**Methods:**

Using QLIPS, which requires only ten minutes of incubation, a cohort of control and SjS sera were tested for antibodies to three SjS autoantigens (La, Ro60 and Ro52). Sensitivity and specificity of the QLIPS tests were compared with LIPS and existing ELISA data. The QLIPS test for Ro52 was then evaluated with a new validation cohort and its diagnostic performance determined.

**Results:**

Using QLIPS, autoantibodies to three SjS antigens, La, Ro60, and Ro52 were detected in 49%, 56% and 70%, respectively, of the SjS patients and none of the controls (100% specificity). With antibody titers in the Ro52-seropositive SjS samples approximately 1,000 times higher than the healthy controls, not only was Ro52 the most informative, but detection of anti-Ro52 antibodies under these non-equilibrium conditions was improved compared to the standard 2 hour LIPS format. Validation of the anti-Ro52 QLIPS test in a new, independent cohort of SjS and control serum samples showed 66% sensitivity and 100% specificity.

**Conclusion:**

Together these results suggest that the QLIPS format for Ro52 yields both a more rapid and more discriminating test for detecting Ro52 autoantibodies than existing immunoassays and has the potential to be adapted for point-of-care evaluation of patients with SjS and other rheumatologic diseases.

## Introduction

There is an increasing desire in the medical community to develop rapid and personalized serum-based diagnostic tests to detect autoimmune [1], neoplastic [2] and infectious diseases [3]. One major approach involves using antibody-based tests to diagnose and even predict the onset of various diseases [1,4]. However, most current quantitative immunoassays used to measure antibodies are impractical for rapid point-of-care testing because they are complex, time consuming, and difficult to standardize [3]. As an alternative, rapid tests such as lateral flow immunoassays, which can more easily be integrated in point of care settings, are used for the diagnosis of several infectious agents such as HIV and HCV. However, one limitation of these assays is that they produce a qualitative (i.e. positive or negative) rather than a quantitative result. Currently there are no serological tests for rapidly detecting autoantibodies associated with autoimmune diseases that also satisfy the growing demand for high analytical sensitivity and reproducibility.

Sjögren's syndrome (SjS) is a common autoimmune disorder associated with epithelial inflammation and exocrine gland dysfunction [5]. SjS is often associated with polyclonal B cell activation resulting in the presence of multiple autoantibodies including the well-known SSA and SSB antibodies. While positive SSA (Ro52 and Ro60) and SSB (La) autoantibodies are part of the diagnostic criteria, five other clinical signs including ocular and oral dryness and evidence of inflammation from minor salivary gland biopsy are required for the diagnosis of primary SjS [6]. This is because antibodies to SSA and SSB are not specific to SjS, but are also found in other rheumatological diseases including systemic lupus erythematosus (SLE), and myositis [7]. Nevertheless, in the 2002 classification standards for SjS diagnosis, positive SSA and SSB autoantibody tests were the only mandatory criteria if the salivary gland biopsy was negative [6]. Current SSA and SSB ELISAs, which employ native antigen complexes isolated from calf thymus, show positive SSA and SSB serology in 50-70% and 40-45% of SjS, respectively [8,9].

Previously, luciferase immunoprecipitation assay systems (LIPS), which employs *Renilla *luciferase (Ruc)-antigen fusions produced in mammalian Cos1 cells, was used to detect patient antibodies to a variety of pathogen antigens [10-17] and also to detect human autoantibodies associated with several autoimmune diseases including Type 1 diabetes [18], Stiff-person syndrome [19] and Sjögren's syndrome [20]. In the SjS studies, detection of anti-La/SSB antibodies by LIPS showed improved performance compared to existing ELISA and offered a highly sensitive, robust and high-throughput testing format [20]. LIPS profiling of additional autoantigens revealed that certain SjS patients also showed positive immunoreactivity with Ro52, Ro60 and other extraglandular autoantigens including thyroid peroxidase, the aquaporin-4 water channel and the gastric H^+^/K^+ ^ATPase.

A quicker version of LIPS (called QLIPS) has also been used to detect antibodies to several pathogen antigens associated with human infection [14,16], in which the two incubation steps of 1 hour were each reduced to 5 minutes. In the present study, we describe QLIPS tests for evaluating antibodies to the 3 major SjS recombinant autoantigens. Results from this study demonstrate that detection of anti-Ro52 antibodies by QLIPS was rapid, robust and has the potential to be used in the diagnosis of SjS and other rheumatologic diseases in point-of-care settings.

## Methods

### Patients

The SjS patients from both cohorts used in this study fulfilled the revised European consensus criteria [6]. The initial cohort of sera was from patients with primary SjS participating in a longitudinal natural history study and was analyzed by the standard LIPS format in a previous study [20]. These sera included 57 well-characterized patients diagnosed with primary SjS and 25 healthy volunteers evaluated under Institutional Review Board-approved protocols at the SjS clinic of the National Institute of Dental and Craniofacial Research, National Institutes of Health, Bethesda, MD. As described, SSA (anti-Ro52 and anti-Ro60 antibodies) and SSB (anti-La antibody) tests on these samples were measured in the Laboratory of Clinical Medicine, NIH using a commercial ELISA obtained from BioRad (Hercules, CA) that employs native, extractable bovine nuclear antigens [20]. The cut-off value used for the ELISA was determined from the internal standards according to the manufacturer.

A second, independent validation cohort collected at the University of Florida under Institutional Review Board-approved protocols consisted of 105 SjS and 30 control sera. For comparison, anti-Ro60 and anti-La (SSB) seropositive status was evaluated in a subset of these samples using a radiobinding immunoprecipitation assay (RBA) [21] in the clinical laboratory of the Division of Rheumatology and Clinical Immunology and Center for Autoimmune Diseases, University of Florida. In these tests, ^35^S-labeled whole K562 cell protein extract was used in immunoprecipitation and following autoradiography was scored positive or negative based on the presence or absence of the La or Ro60 immunoprecipitated protein.

### Renilla luciferase antigen constructs and extracts

A mammalian *Renilla *luciferase (Ruc) expression vector, pREN2 [22], expressing Ruc-antigen fusion constructs for La, Ro52, Ro52-Δ2 (spanning amino acid residues 278-475) and Ro60, has been previously described [20]. In the case of Ro60, a new pREN2 construct (Ro60-Δ2) expressing a C-terminal protein fragment spanning amino acid residues 336-576, was generated. DNA sequencing confirmed the integrity of this Ro60-Δ2 plasmid construct.

Cos1 cells in 100 mm^2 ^dishes were transfected with Ruc-antigen plasmids and lysates prepared as described [22]. Briefly, Cos1 cells in 100 mm^2 ^dishes were transfected using FuGENE 6 (Roche) with 1-2 μg of pREN2 plasmid constructs. Forty-eight hours after transfection, tissue culture media was removed and the plates were washed with PBS. The cells were then scrapped in 1.4 ml of cold lysis buffer composed of 50 mM Tris, pH 7.5, 100 mM NaCl, 5 mM MgCl_2_, 1% Triton X-100, 50% glycerol and protease inhibitors (Mini protease inhibitor cocktail, Roche). The cell lysate was sonicated, centrifuged and the cleared supernatants were collected and used immediately or stored at -80°C. Total luciferase activity in 1 μl of each crude extract was determined by adding it to 9 μl of PBS in a 1.5 ml clear microfuge tube, followed by the addition of 100 μl of substrate mixture (*Renilla *Luciferase Reagent Kit, Promega), vortexing, and immediately measuring light-forming units with a luminometer (20/20^n ^Turner Scientific) for 5 sec.

### QLIPS

A shortened version of LIPS designated QLIPS (for quick LIPS) was employed [14,16]. In these assays, sera were processed in a 96-well format. A "master plate" was first constructed by diluting patient sera 1:10 in assay buffer A (50 mM Tris, pH 7.5, 100 mM NaCl, 5 mM MgCl_2_, 1% Triton X-100) in a 96-well polypropylene microtiter plate. For evaluating antibody titers by LIPS, 40 μl of buffer A, 10 μl of diluted human sera (1 μl equivalent), and 1 × 10^7 ^light units (LU) of Ruc-antigen Cos1 cell extract, diluted in buffer A to a volume of 50 μl, were added to each well of a polypropylene plate and incubated for 5 minutes at room temperature with shaking. Next, 5 μl of a 30% suspension of Ultralink protein A/G beads (Pierce Biotechnology, Rockford, IL) in PBS were added to the bottom of each well of a 96-well filter HTS plate (Millipore, Bedford, MA). To this filter plate, the 100-μl antigen-antibody reaction mixture was transferred and incubated for 5 minutes at room temperature on a rotary shaker. The washing steps of the retained protein A/G beads were performed on a BioMek FX work station (Beckman Coulter, Fullerton, CA) using an integrated vacuum manifold. For these washes, each well is washed 8 times with 100 μl of buffer A, followed by two times with 100 μl of PBS. After the final wash, the filter plate is blotted dry and LU were measured in a Berthold LB 960 Centro microplate luminometer (Berthold Technologies, Bad Wilbad, Germany) using coelenterazine substrate mix (Promega, Madison, WI). For these measurements, 50 μl of coelenterazine substrate is injected, the plate is shaken for 2 sec, followed by a 5 sec read of luminescence. All LU data were obtained from the average of at least two independent experiments, and the resulting LU values were used without subtracting the buffer blank.

### Statistical analysis

The GraphPad Prism software (San Diego, CA) was used for statistical analyses. Results for quantitative antibody levels of the controls and SjS serum samples are reported as the geometric mean titer (GMT) ± 95% confidence interval (due to the typically overdispersed nature of these data). Correlations among antibody responses to the antigens tested were assessed by the Spearman rank test (*r*_*S*_). The level of statistical significance for all tests was set at *P *< 0.05. For determining the cut-off limits for each of the QLIPS tests, the mean value of the 25 control samples plus 5 SD in the first cohort was used and is indicated in the figures. Additional analysis using a cut-off derived from the mean plus 3 SD is also included in the text. Test performance was evaluated using area under the curve (AUC) from receiver operator characteristic (ROC) analysis.

## Results

### Detection of anti-La autoantibodies in SjS by QLIPS

**Figure 1:**
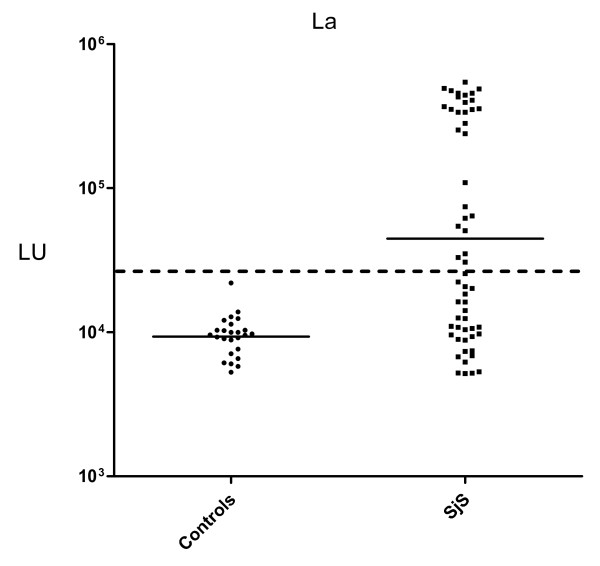
**QLIPS detection of anti-La autoantibodies**. QLIPS detection of autoantibodies against La in 25 normal controls and 57 primary SjS patients. Each circle or square symbol represents the anti-La antibody titer of a normal control or SjS patient, respectively. The solid lines represent the GMT for each group. For determining sensitivity and specificity for this anti-La antibody test, the dashed line represents the cut-off level derived from mean plus 5 SD of the antibody titers of the 25 normal volunteers.

The diagnostic performance of a previously described QLIPS format was evaluated for measuring autoantibodies to the three major SjS antigens. From testing a cohort of 57 SjS and 25 healthy control sera with a full-length La recombinant fusion protein, the geometric mean titer (GMT) of anti-La antibodies was 44,692 LU (95% CI, 28,604-69,827) for SjS sera, which was 4 times higher than the GMT of the control sera of 9,156 LU (95% CI, 8,097-10,355) (Figure 1). Compared to our previously published study [20], the results using the QLIPS format showed anti-La antibody titers that were ~10-fold lower than the anti-La antibody titers reported by the standard 2 hour incubation format (data not shown). Nevertheless, evaluation by the Mann Whitney *U *test still showed a significant difference between the anti-La antibody titers in the SjS samples and the controls (*P *< 0.0001). To examine the diagnostic utility of the anti-La QLIPS test, the sensitivity and specificity were determined. For this calculation, a cut-off value of 26,869 LU derived from the mean plus 5 SD of the 25 control samples was used. Based on this cut-off, the anti-La antibody test showed 49% sensitivity and 100% specificity in distinguishing the 57 SjS sera from the 25 control sera. An even lower cut-off derived from the mean plus 3 SD yielded 56% sensitivity and 96% specificity. While these QLIPS results for detecting anti-La antibodies were similar to an established ELISA test for anti-SSB/La antibodies (46% sensitivity), this shortened assay was not as useful as the our previous LIPS results on these same samples with 75% sensitivity and 100% specificity [20]. Finally, the results from the duplicate interassay QLIPS tests for anti-La antibodies showed that they were reproducible and had a coefficient of variation of 18.9%.

### Rapid detection of anti-Ro60 and anti-Ro52 autoantibodies in SjS by QLIPS

**Figure 2:**
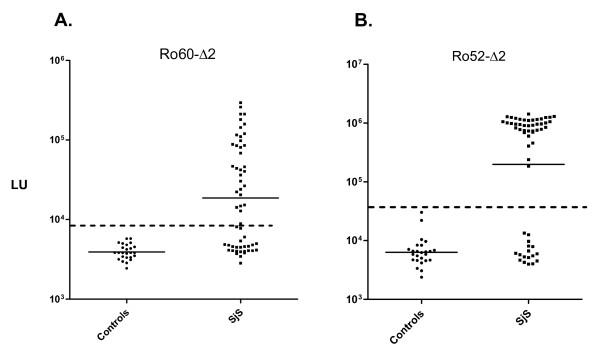
**QLIPS detection of anti-Ro60 and anti-Ro52 autoantibodies in SjS**. QLIPS detection of autoantibodies against Ro60-Δ2 and a C-terminal fragment of Ro52 (Ro52-Δ2) in 25 normal volunteers and 57 primary SjS patients. (A) The anti-Ro60-Δ2 antibody test. (B) The anti-Ro52-Δ2 antibody test. Each circle or square symbol represents individual normal controls or SjS patient samples, respectively. The solid lines represent the GMT for each group. For determining sensitivity and specificity, the dashed line represents the cut-off level derived from the control mean plus 5 SD.

We have previously reported using a full-length Ro60-Ruc antigen fusion in the LIPS format, which required a cumbersome 1:200 dilution of human sera to place detection of anti-Ro60 autoantibodies in the linear range [20]. In order to simplify testing, a C-terminal Ro60 deletion fragment (Ro60-Δ2) was generated and found to yield values in the linear range without the need to dilute the sera. As shown in Figure 2A, testing of this Ruc-Ro60-Δ2 fusion by QLIPS revealed that the GMT of the anti-Ro60 antibody in the 57 SjS samples was 18,967 LU (95% CI, 12,659-27,613), which was over 4-fold higher than the GMT of 3,917 LU (95% CI, 3,574-4293) of the controls (Mann Whitney *U *test, *P *< 0.0005). The anti-Ro60-Δ2 antibody titers detected by QLIPS were ~10-fold lower compared to when the same sera were tested in the standard 2 hour incubation LIPS format (data not shown). Calculations of the diagnostic performance of the Ro60-Δ2 QLIPS test based on the mean plus 5 SD of the 25 control samples (i.e. 8,466 LU) showed 56% sensitivity and 100% specificity in distinguishing the 57 SjS sera from the 25 control sera. An even lower cut-off derived from the mean plus 3 SD yielded 60% sensitivity and 100% specificity.

QLIPS testing for anti-Ro52 autoantibodies using a C-terminal fragment (Ro52-Δ2) showed that the GMT of the SjS sera was 198,110 LU (95% CI, 107,237-365,988), which was 30-times higher than the GMT of the control sera of 6,351 LU (95% CI, 5,068-7,957) (Figure 2B). The results from the duplicate interassay QLIPS tests for anti-Ro52-Δ2 antibodies were reproducible and had a coefficient of variation of 19%. To examine the diagnostic utility of Ro52-Δ2, the sensitivity and specificity of the QLIPS test were calculated using a cut-off value (i.e. 37,806 LU) derived from the mean plus 5 SD of the 25 control samples. Using this cut-off, the Ro52-Δ2 antibody test showed 70% sensitivity and 100% specificity in distinguishing the 57 SjS patient from controls. An even lower cut-off derived from the mean plus 3 SD still showed 70% sensitivity and 96% specificity. Among the 40 Ro52-Δ2 seropositive SjS samples, the Ro52-Δ2 antibody titers were approximately 1,000 times higher than the controls or Ro52-Δ2 seronegative SjS samples.

Interestingly, the Ro52-Δ2 QLIPS test showed *higher *sensitivity and specificity than the standard 2 hour LIPS format on the same samples[20], which was only 65% sensitive and 96% specific. Additional analysis revealed that the antibody values in the 57 SjS samples by QLIPS and LIPS were similar and correlated well (*r*_*s *_= 0.72). For example, the mean and standard deviation in the 57 SJS samples by QLIPS and LIPS were similar with values of 650,273 ± 485, 490 and 548,504 ± 398,083 LU, respectively. In contrast, the antibody titer values for the 25 control samples showed a mean and standard deviation by QLIPS and LIPS of 7,576 ± 6,046 and 29,770 ± 78,495 LU, respectively. Of note, the markedly higher average and standard deviation of the control group measured by LIPS were due to several high titer outliers among the control samples, which were likely due to low affinity antibodies since they disappeared in the QLIPS format. While all the LIPS Ro52 positives were also positive by QLIPS, 2 samples that were negative by LIPS were now positive by QLIPS. The net result of these high titer controls in the LIPS format was a much higher cut-off with an AUC value of 0.83 for test performance. The AUC value for QLIPS test was 0.85 and was slightly higher than LIPS reflecting the higher sensitivity and specificity.

Based on these findings, the most informative autoantigen in the QLIPS test for SjS was Ro52-Δ2 with 70% sensitivity and 100% specificity. Including the results from the anti-La and anti-Ro60 QLIPS tests did not add any new positives to the existing Ro52-Δ2 QLIPS test. Performance of the SSA ELISA on the same samples, which measures antibodies to both Ro52 and Ro60 proteins, had 72% sensitivity but included 3 ELISA borderline positive cases. These results suggest that QLIPS Ro52-Δ2 test shows similar sensitivity to an ELISA, but produces quicker and more robust results.

### The Ro52-Δ2 QLIPS test in a new SjS cohort shows 66% sensitivity and 100% specificity

**Figure 3:**
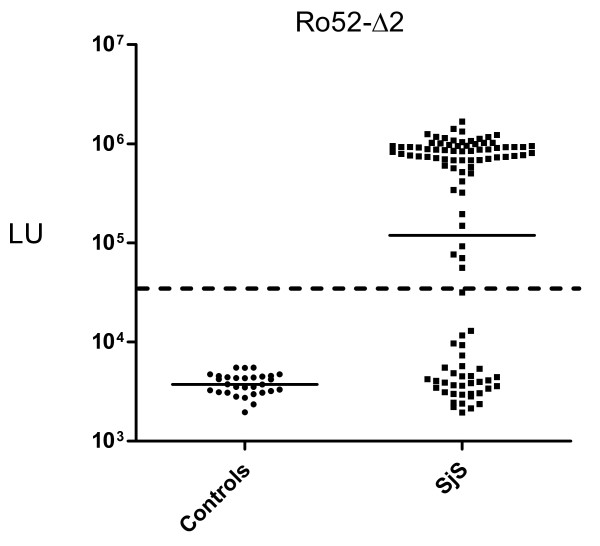
**Ro52-Δ2 QLIPS testing with an independent validation cohort**. Antibodies were evaluated by QLIPS with 107 SjS and 30 control sera. Each circle or square symbol represents individual normal controls or SjS patient samples, respectively. The solid lines represent the GMT for each group. The cutoff of 37,806 LU, previously determined from the initial cohort in Figure 2B, is shown by the dotted line and results in 66% sensitivity and 100% specificity.

A new, independent validation cohort of 105 SjS and 30 control sera was tested to verify the diagnostic utility of the Ro52-Δ2 QLIPS test. Robust signals were detected in the SjS samples from this validation cohort, in which the GMT in the SjS samples was 119,092 LU (95% CI, 72,924-194,489), and the GMT of the controls was 3,735 LU (95% CI, 3,400-4,102). Comparison of the anti-Ro52-Δ2 antibody data plots between the initial and validation cohorts show that they are remarkably similar (Figure 2B vs. Figure 3), in which the mean LU values for Ro52 antibody titers in the initial and validation cohort are almost identical with values of 650,273 and 529,711 LU, respectively. Using the previous cut-off of 37,806 LU, the QLIPS test distinguished 69 of the 104 SjS positive samples (66% sensitivity) from the 30 controls with 100% specificity (Figure 3). If a cut-off derived from the controls of the validation cohort (i.e. 9,000 LU) is used, an even higher sensitivity of 70% is achieved, while still maintaining 100% specificity. Furthermore, compared to RBA for anti-Ro60 and anti-La antibodies performed on the same validation samples, the QLIPS test for Ro52 had a significantly higher sensitivity (66% versus 56%).

## Discussion

Rapid and comprehensive serum-based diagnostic tests that can be used in point-of-care settings for diagnosis and even pre-symptom screening of autoimmunity are urgently needed. A significant challenge in the development of such assays is that, unlike antibodies associated with infectious agents, the detection of autoantibodies associated with autoimmunity requires more sensitive tests than ELISAs or other solid phase immunoassays such as protein arrays, which miss many conformational epitopes [23]. Typically, liquid phase immunoprecipitation assays such as the radiobinding assay (RBA), which show much higher sensitivity, specificity and signal to noise ratios than ELISAs are needed for detecting autoantibodies in most autoimmune diseases [23,24]. However, a significant drawback of RBAs is the requirement for radioactively-tagged antigens. In this study we demonstrate that QLIPS, which utilizes a non-radioactive, luciferase enzyme-based tracer in a liquid phase assay, can rapidly and sensitively detect autoantibodies associated with SjS. The QLIPS format could easily be integrated into a point-of-care test because it requires only about 25 minutes of total processing time per 94 sera samples, which includes a 5 minute set-up, two five minute incubations steps, 10 minutes of washing and reading of the plate with a luminometer.

Due to the high signal to noise and large dynamic range of the LIPS assay, the coefficient of variation (CV) of approximately 20% for LIPS still provides remarkable diagnostic accuracy. One likely cause of the near 20% CV is due to the fact that the QLIPS sample processing (i.e. pipetting and washing) was preformed rapidly in less than 15 minutes with 84 or greater samples. With some of these high signals in the SjS positive samples, small pipetting errors can resulting in large changes in antibody titer (e.g. 10% pipetting error can result in over 100,000 LU differences). Despite the 20% CV, the SjS positive samples show 1000-fold higher anti-Ro52 antibody titers than the negative samples and evaluation of each of the two runs independently shows that show that the same samples are positive.

Previously, we found that all individuals have detectable anti-Ro52 antibodies by LIPS [20]. However, unlike the normal range of anti-Ro52 antibody titers in healthy individuals, some patients with SjS or other rheumatological diseases have markedly higher anti-Ro52 antibody titers that can be detected by LIPS and other immunoassays. In this study, the QLIPS test for anti-Ro52-Δ2 antibodies had a higher diagnostic performance than the standard LIPS format. The reason for this increased performance is due to the loss of anomalously high signals in some of the control samples observed in the standard LIPS format. These anomalously high Ro52 antibody signals in several of the controls were no longer positive under the rapid, non-equilibrium conditions of QLIPS. Taken together these results also suggest that performing QLIPS and LIPS in parallel may allow a simple method of more accurately assessing antibody avidity in some situations. An analogous increase in specificity of QLIPS compared to LIPS has also been observed for distinguishing antibodies to *Loa loa *and *Onchocerca volvulus *antigenic proteins from antibodies to antigens from related filarial infections [14,16]. In contrast, the QLIPS tests for anti-La and anti-Ro60 antibodies showed a marked drop in test performance in the QLIPS format. For example, the LIPS test for detecting anti-La antibodies was 75% sensitive, versus the 49% sensitivity of QLIPS. The decreased detection of anti-La and anti-Ro60 seropositive antibodies under these rapid conditions compared to LIPS is likely due in part to the inability to detect the low affinity/low titer autoantibodies present in some of the SjS samples. However, we show that using QLIPS, the SjS patients can be distinguished from controls using the Ro52-Δ2 fragment alone.

While the standard LIPS format yielded 76% sensitivity and required two independent assays (anti-La and anti-Ro52 autoantibodies) to be performed [20], QLIPS, with a single antigenic fragment of Ro52, showed approximately 70% sensitivity. Ironically, this C-terminal fragment of Ro52 used in QLIPS is the same antigenic fragment that shows no useful diagnostic immunoreactivity in ELISA and Western blotting [25,26]. The detection of diagnostically useful antibodies to the C-terminus of Ro52 by LIPS is supportive of the improved conformational epitopes using mammalian recombinant proteins in this liquid phase QLIPS/LIPS compared to ELISA. Both the LIPS and QLIPS formats are also as good as a conventional ELISA for measuring SSA and SSB. However, an ELISA requires significantly more time to complete (e.g. 5-24 hours). Furthermore, the QLIPS Ro52 test showed higher sensitivity in the validation cohort than an established RBA for SSA and SSB (66% vs. 56%).

The short incubation time, high performance and relative simplicity of the Ro52-Δ2 QLIPS test has practical implications for developing even simpler assay formats. The finding that the Ro52-Δ2 QLIPS test showed antibody titers that were 1000 times higher in the SjS positive samples compared to the control samples also provides a large diagnostic window for detecting seropositive samples. Furthermore, no other immunoassay format such as ELISA or RBA shows such a large signal-to-noise ratio. It is likely that additional assay modification, including reducing the volume of the reaction, may yield even more robust signals. We speculate that a microfluidic device configured for the QLIPS format might be suitable for point-of-care testing. Using such a microfluidics device, the addition of sera, Ruc-antigen mixture to immobilized protein A/G, washing, and the addition of coelenterazine luciferase substrate could all be automated and performed rapidly. The ability to stably freeze the Ruc-antigen reagents also has practical implications for point-of-care testing. Due to the highly scalable format of QLIPS, additional reagents for detecting anti-pathogen antibodies (e.g. HIV, HCV, and HSV-2) could also be employed for side-by-side diagnosis of these infections.

## Conclusion

Ro52 autoantibodies are not only found in SjS, but are found in SLE, myositis and several other autoimmune disorders. These results suggest that the rapid and robust Ro52-Δ2 QLIPS test has the potential to aid in point-of-care evaluation of patients with SjS and other rheumatologic diseases. However, since not all SjS patients show anti-Ro52 positive antibodies, the addition of other autoantigens might improve the diagnostic performance of the QLIPS test. In particular, antigens that produce robust signals in Ro52-negative sera might be part of an antigen mixture used in the QLIPS format to further increase the sensitivity of this test. The ability of using QLIPS for screening for anti-Ro52 and other autoantibodies in early phases of the disease might make it possible to diagnose and even treat autoimmune diseases before more severe disease and/or substantial organ damage has occurred.

## Competing interests

Two of the authors (P.D.B., and M.J.I.) have a patent application submitted using LIPS for detecting autoantibodies associated with Sjögren's syndrome.

## Authors' contributions

PDB conceived of the study, developed the needed constructs, analyzed the sera by LIPS, analyzed the data, drafted the manuscript and made critical revisions; ATI, KHC and CL generated the needed constructs and/or lysates; YL and MS analyzed the sera by conventional immunoprecipitation assays; WHR, provided patient sera from cohort 2 with clinical information and was involved in critical revision; MJI helped develop the high-throughput assay and was involved in critical revision and final approval and all authors commented on and approved the manuscript.
